# Supplementary material for: LFP beta amplitude is linked to mesoscopic spatio-temporal phase patterns
Source: Sci Rep. 2018 Mar 26;8:5200. doi: 10.1038/s41598-018-22990-7 (PMC5980111; doi:10.1038/s41598-018-22990-7)
Supplement: Supplementary file 1 — Supplementary Figures [file 41598_2018_22990_MOESM1_ESM.pdf]

Supplemental Figures:
LFP beta amplitude is linked to mesoscopic spatio-temporal phase patterns

Michael Denker[1,*], Lyuba Zehl[1], Bjørg E. Kilavik[2], Markus Diesmann[1], Thomas Brochier[2], Alexa Riehle[2,1,3], and Sonja Grün[1,3,4]

[1]Institute of Neuroscience and Medicine (INM-6) and Institute for Advanced Simulation (IAS-6) and JARA-Institute Brain Structure Function Relationship (JBI 1 / INM-10), Jülich, Germany
[2]Institut de Neurosciences de la Timone (INT), CNRS-Aix-Marseille University, UMR 7289, Marseille, France
[3]RIKEN Brain Science Institute, Wako City, Japan
[4]Theoretical Systems Neurobiology, RWTH Aachen University, Germany
[*]Correspondence to: m.denker@fz-juelich.de

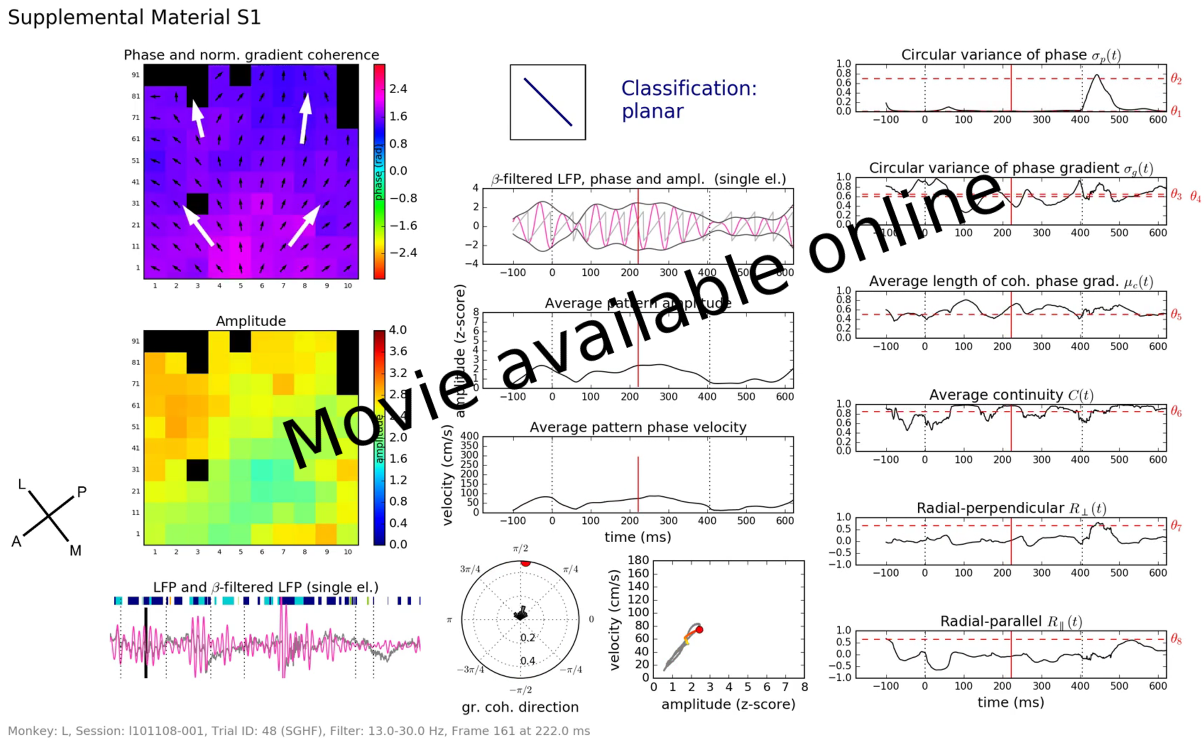

Movie S1. Movie depicting the formation of wave patterns over time (available online).

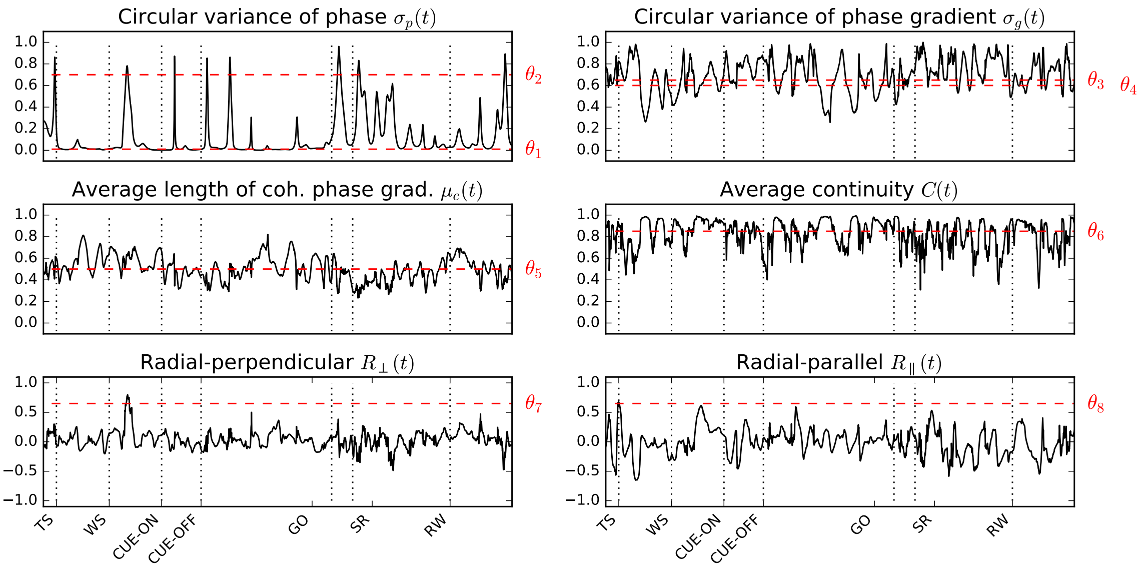

Figure S2. Thresholds used for automatic classification of the example trial. Graphs of the time series (corresponding to the trial shown in panel Figure 3A) of the 6 measures used for detection of the different patterns (top to bottom: $\sigma_p(t)$, $\sigma_g(t)$, $\mu_c(t)$, $C(t)$, $R_\parallel$, and $R_\perp$). Red dashed lines indicate the thresholds θ_1 to θ_8 used to detect and classify the phase patterns at each time point.

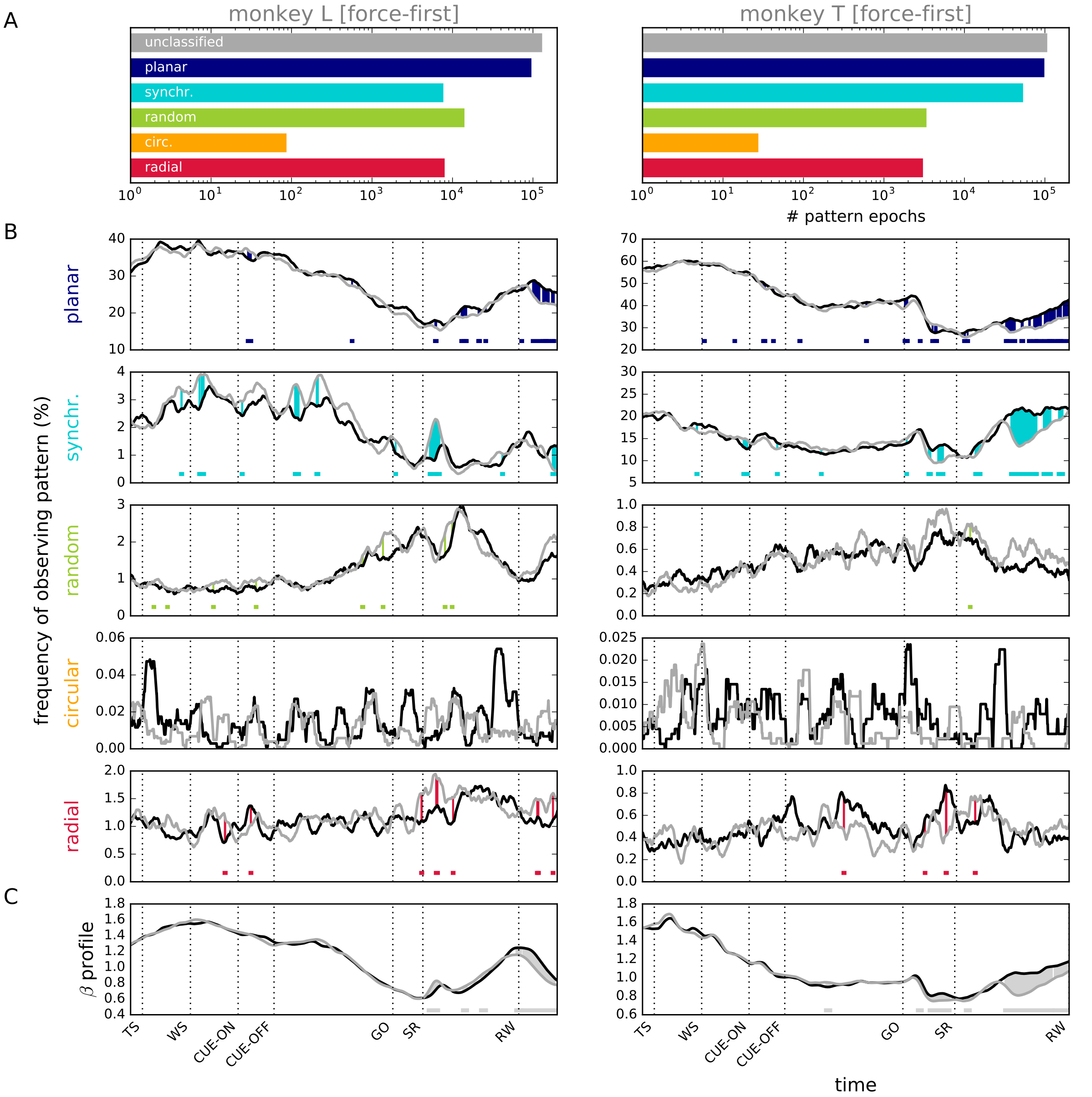

Figure S3. **Behavioral correlates and relation to average beta amplitude for the force-first condition.**. **A.** Number of epochs of a phase pattern detected in at least 5 consecutive time frames, i.e., 5 ms (bars from top to bottom: unclassified, planar wave, synchronized, random, circular, radial pattern) for monkey L (left) and T (right). Data were obtained from all selected recording sessions including inter-trial intervals. **B.** Time-resolved probability of observing a specific phase pattern (rows) during the trial. Statistics were computed across all force-first trials of all recording sessions for all monkeys ($N = 15$) and smoothed with a box-car kernel of length $l = 100$ ms. Trials were separated into side-grip (SG) trials (black) and precision-grip (PG) trials (gray). In contrast to Figure 4, differences between these trial types occur only after the grip information presented at GO. Color shading between curves and colored bars indicate time periods where SG and PG curves different significantly (Fisher's exact test under the null hypothesis that, for any time point, the likelihood to observe a given phase pattern is independent of the trial type, $p < 0.05$). **C.** Beta amplitude profile (envelope) pooled across all SG (black) and PG (gray) trials (same data as in panel B). The amplitude profile $a(t)$ of a single trial is calculated as the time-resolved instantaneous amplitude $A_{xy}(t)$ of the beta-filtered LFP averaged across all electrodes (x, y), and measures the instantaneous power of the beta oscillation in that trial. Gray shading between curves and horizontal bars indicate time periods where SG and PG curves differ significantly (t-test under the null hypothesis that the distributions of electrode-averaged single trial amplitudes $A_{xy}(t)$ at each time point t are identical for SG and PG trials, respectively, $p < 0.05$).

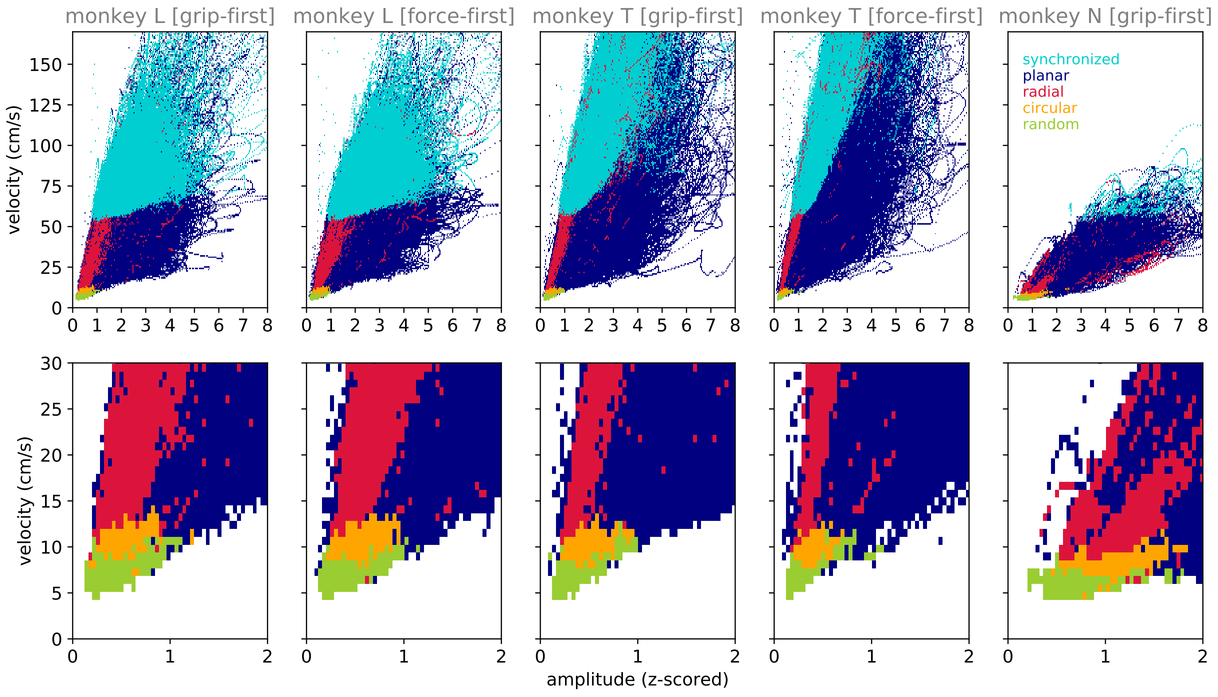

Figure S4. Regions of dominant phase patterns as a function of amplitude and velocity. Upper row: For each monkey and behavioral condition (columns), at each value of the phase velocity $v(t)$ and beta amplitude profile $a(t)$ (400 bins on each axis), the graph indicates by color the most frequently observed phase pattern relative to the total number of time bins in which each respective phase pattern class was observed (i.e., independent of overall likelihood to observe a particular pattern class). White areas indicate that at the corresponding combination of amplitude and velocity no phase pattern was observed in the data. Lower row: Zoom-in for small amplitudes and velocities.

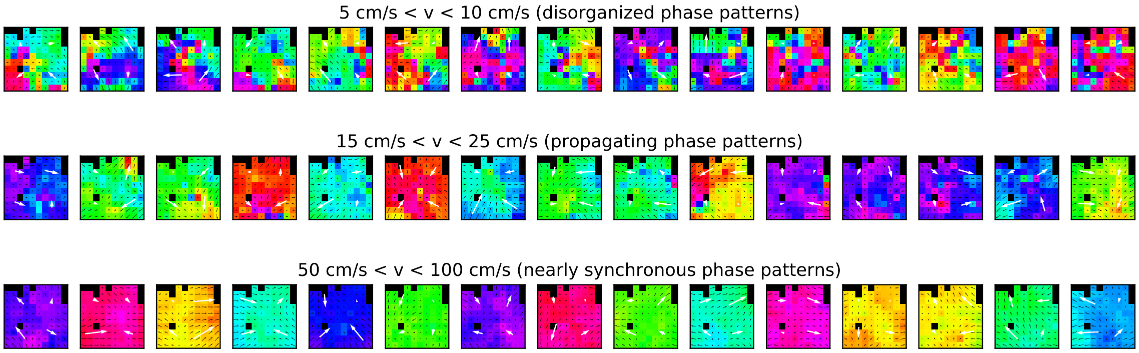

Figure S5. **Examples of unclassified phase patterns as a function of velocity.** Each row shows 15 randomly drawn examples of phase patterns that were marked unclassified due to their ambiguity, grouped by the velocity measure calculated from the corresponding phase map: $5-10$ cm/s (top), $15-25$ cm/s (middle), and $50-100$ cm/s (bottom). Despite of the ambiguity, velocity distinguishes seemingly disorganized patterns at low velocities similar to random and circular patterns, patterns exhibiting a propagation (intermediate velocities) reminiscent of planar and radial patterns, and nearly synchronized patterns (high velocities). Examples sampled from recording l101108-001 (monkey L).

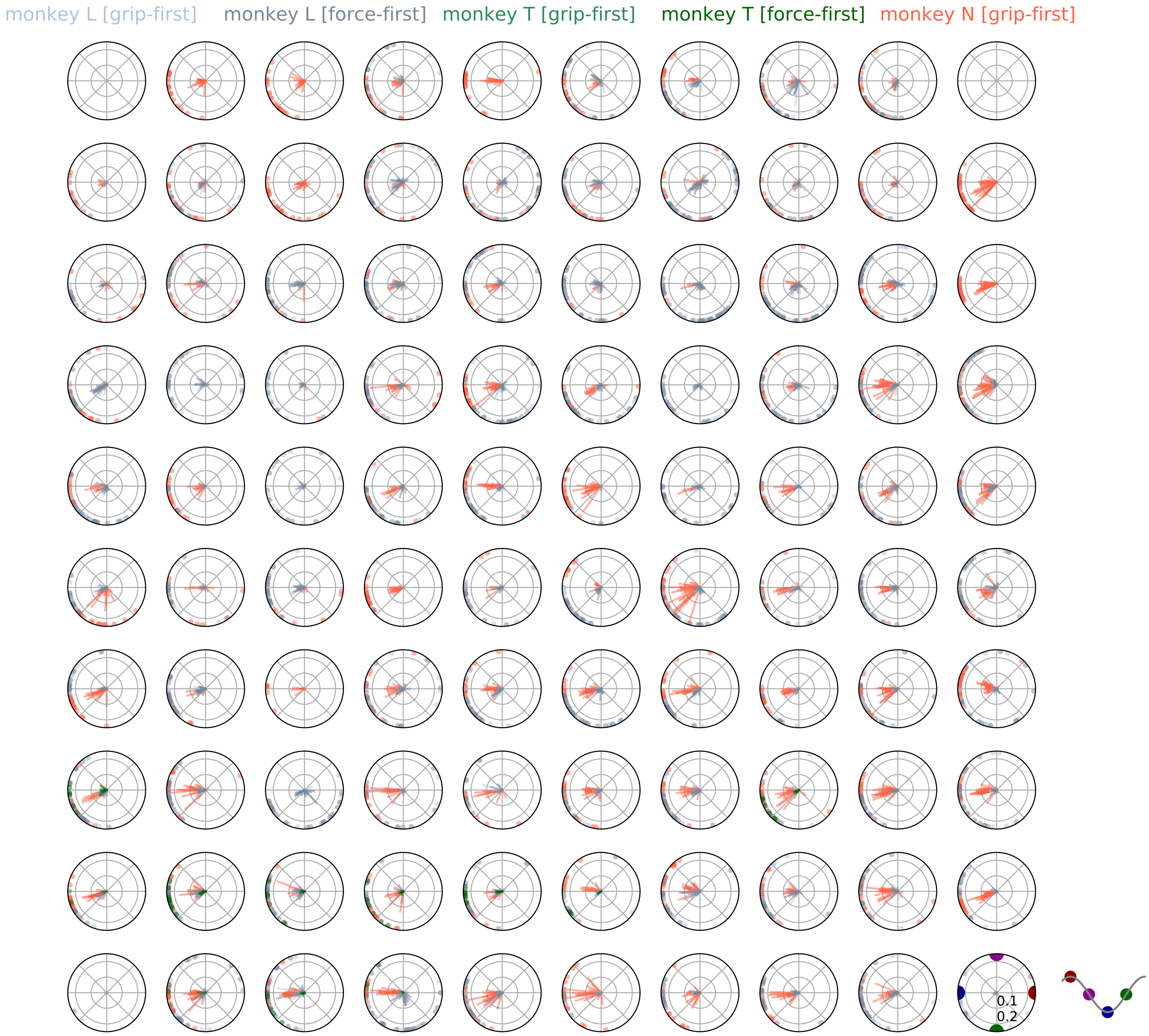

Figure S6. **Coupling of spiking activity to beta oscillations.** Locking strength R (line lengths) and average phase ϕ (line angles and dots on the outline of the polar plots) of phase locking of single units to the beta-filtered LFP at each electrode of the array. The polar plots are arranged in a 10x10 grid representing the electrodes of the array as depicted in Figure 3. The phase locking value of one single unit was calculated as the mean phase of spike-triggered LFP phases: $R \exp i\phi = \sum \exp i\phi(t_i)$, where $\phi(t_i)$ indicates the beta phase at spike time t_i. The color of lines and dots indicate the monkey and experimental condition of the recording session the single unit was recorded from (see legend on top). In total, for the grip-first condition we analyzed $N = 1044$, $N = 9$, and $N = 1704$ single units for monkeys L, T, and N, and for the force-first conditions $N = 1041$ and $N = 50$ single units for monkeys L and T, respectively. Single-units were sorted offline using the Plexon Offline Spike Sorter (Plexon Inc, Dallas, Texas, USA, version 3.3). The mapping of the polar coordinate system to the LFP oscillation cycle is sketched in the lower right.
